# Improving quality through simulation; developing guidance to design simulation interventions following key events in healthcare

**DOI:** 10.1186/s41077-024-00300-8

**Published:** 2024-07-17

**Authors:** Cristina Diaz-Navarro, Bridie Jones, Gethin Pugh, Michael Moneypenny, Marc Lazarovici, David J. Grant

**Affiliations:** 1 Health Education and Improvement Wales, Cardiff, UK; 2 Cardiff and Vale University Health Board, Cardiff, UK; 3 Improvement Cymru Academy, Cardiff, UK; 4 Clinical Skills Managed Educational Network, NHS Education for Scotland, Dundee, UK; 5 Association for Simulated Practice in Healthcare, Lichfield, UK; 6 Institut Für Notfallmedizin Und Medizinmanagement (INM), LMU University Hospital, Munich, Germany; 7 SESAM - Society for Simulation in Europe, Munich, Germany; 8 University Hospitals Bristol and Weston NHS Foundation Trust, Bristol, UK; 9 Faculty of Health Sciences, University of Bristol Medical School, Bristol, UK

**Keywords:** Quality improvement, Simulation, Key event, Critical incident, Patient safety

## Abstract

Simulation educators are often requested to provide multidisciplinary and/or interprofessional simulation training in response to critical incidents. Current perspectives on patient safety focus on learning from failure, success and everyday variation. An international collaboration has led to the development of an accessible and practical framework to guide the implementation of appropriate simulation-based responses to clinical events, integrating quality improvement, simulation and patient safety methodologies to design appropriate and impactful responses. In this article, we describe a novel five-step approach to planning simulation-based interventions after any events that might prompt simulation-based learning in healthcare environments. This approach guides teams to identify pertinent events in healthcare, involve relevant stakeholders, agree on appropriate change interventions, elicit how simulation can contribute to them and share the learning without aggravating the second victim phenomenon. The framework is underpinned by Deming’s System of Profound Knowledge, the Model for Improvement and translational simulation. It aligns with contemporary socio-technical models in healthcare, by emphasising the role of clinical teams in designing adaptation and change for improvement, as well as encouraging collaborations to enhance patient safety in healthcare. For teams to achieve this adaptive capacity that realises organisational goals of continuous learning and improvement requires the breaking down of historical silos through the creation of an infrastructure that formalises relationships between service delivery, safety management, quality improvement and education. This creates opportunities to learn by design, rather than chance, whilst striving to close gaps between work as imagined and work as done.

Simulation educators are often requested to provide multidisciplinary and/or multi-professional simulation training in response to a critical incident that has resulted in a significant adverse event. This raises the question: Should we adopt a systematic approach to designing simulation-based interventions after key events in healthcare environments?

“Simulation is a learning tool that supports development through experiential learning by creating or replicating a particular set of conditions which resemble real life situations; it should provide a safe environment where participants can learn from their mistakes without any danger to patients, allowing individuals to analyse and respond to these realistic situations, with the aim of developing or enhancing their knowledge, skills, behaviours and attitudes” [1]. Simulation-based education and training has a crucial role in improving the quality and safety of care for patients [2]; however, we should not assume that training will solve all issues surrounding critical incidents. Simulation not only provides opportunities for training and debriefing on safety behaviours, but can also play a significant role in improving healthcare systems [3].

A collaboration between Health Education and Improvement Wales (HEIW), Improvement Cymru, the Association for Simulation Practice in Healthcare (ASPiH), the Society for Simulation in Europe (SESAM) and international simulation experts was established. Its purpose was to design an accessible and practical framework to guide implementation of appropriate simulation-based responses following key clinical events, utilising quality improvement (QI) methodology to inform simulation design. The intended outcome is that when simulation is considered in the response to key events, it is used for the right reasons and in the most beneficial way whilst contributing to wider processes that support improvement.

This framework aims to be useful to anyone in the health and care community that wishes to engage in developing simulation-based responses following an event. Notably, it emphasises the importance of including individuals with simulation expertise within the team designing the response.

## Development process

A literature search was carried out on different databases including Medline, Embase, Cumulated Index of Nursing & Allied Health Literature, Educational Resources Information Centre and Scopus. The search focused on simulation (including augmented reality and virtual reality), patient safety, quality improvement and error or event. A total of 113 articles were identified, none of which provided broad guidance on how to design simulation activities following a key event in healthcare.

A multi-professional working group was then established within NHS Wales in order to identify relevant needs and priorities when designing simulation interventions following key events. This group included patient safety leads, simulation educators, quality improvement experts, clinicians and managers. Discussions held in this group highlighted the need to develop guidance based on robust quality improvement methodology and translated into a practical “step-by-step” framework.

An advisory group was set up engaging national and international simulation and QI leads, to review the concept and its applicability, and further develop the content. A panel of six international experts provided iterative peer review, both after the initial concept design and after the document was finalised. The draft framework was trialled during a conference workshop where 32 multi-professional potential users were divided into four groups and asked to produce an initial design of a simulation-based response to a learning event. During the 90-min session, participants completed the allocated task successfully and their feedback contributed to the latest iteration of the document.

The framework, based on QI principles, provides a step-by-step guide to the design of simulation-based interventions in order to improve patient safety (Table 1). It describes how to incorporate best practice, signposting to relevant resources and guidance for intervention evaluation.

**Table 1:** Summary of the 5 steps to design simulation interventions following events in healthcare

What is a key event?	Any circumstance that triggers the consideration of simulation to promote learning and improvement. This includes the introduction of new processes, challenging clinical situations with positive outcomes, near misses or critical events
Who needs to be involved?	What system are we trying to understand? What teams/professions are part of this system? What teams can contribute to the design or delivery of the intervention? What teams/individuals can contribute to making the intervention successful? How will this intervention affect each of them?
What needs to be done?	What specific issue needs addressing? Process mapping (including simulation) What are we trying to accomplish? How will we know that a change is an improvement? What changes can we make that will result in an improvement?
How will simulation contribute?	Potential roles of the simulation-based contribution: System testing Education and training Event debriefing Also consider: staff wellbeing, blended learning approach and iterative evaluation
How should we share the learning?	We can learn from all stages. This learning must be fed back and disseminated adhering to SQUIRE 2.0 reporting guidance

The QI philosophy underpinning the framework is Deming’s System of Profound Knowledge. He described the foundations of improvement science and focused on identifying ways to define the essential components, interactions and variation within systems [4], hence promoting transformation through an essential outside “lens” which can benefit anyone and any organisation. We also refer to well recognised and commonly used QI approaches such as the Model for Improvement [5] and Plan-Do-Study-Act (PDSA) cycles [6].

Other approaches bolstering the framework include the psychology of change [7], which describes key elements necessary to engage people in co-designing improvement; translational simulation [8], which explores how simulation can improve patient outcomes; safety II [9], which changes our learning focus exclusively from risk and failure and widens it to understand the determinants of success and performance variability; and the description of healthcare socio-technical systems provided by the Systems Engineering Initiative for Patient Safety (SEIPS) [10].

This five-step framework aims to be useful across the health and care community to guide the development of simulation responses following a key event.

## The 5 steps

### What is a key event in healthcare?

Current perspectives on patient safety have shifted from focussing on learning from risk and failure (safety I) to understanding learning from failure, success and everyday variation (safety II) [9]. Therefore, we define key events as any circumstance that might trigger the consideration of simulation to promote learning and improvement in a proactive or reactive fashion, including mission rehearsal during introduction of new processes, challenging clinical situations with positive outcomes, near misses or critical events. Examples of key events include simulation-based testing of a new patient care pathway or simulation to reinforce positive behaviours following a well-managed major haemorrhage. The key event becomes a driver for learning and change, contributing to workforce engagement in patient centred system design and improvement.

### Who needs to be involved in the process?

Healthcare systems are inherently complex, and a collaborative approach has the benefit of providing multiple perspectives on a given system, leading to a better understanding of all processes, their connections and impact of potential changes. All individuals who are part of the system under review, and those taking part in the intervention (including teams with expertise in simulation and quality improvement), should be identified to facilitate better understanding of the problem and the design of future improvements. These might include doctors, nurses, allied healthcare professionals, ancillary staff, administrative staff, managers, patients, user groups and educators. A stakeholder analysis must identify these people before the intervention is designed. Its potential impact on all individuals and teams involved is explored, including groups who may be negatively affected as a result of the intervention or change.

### What needs to be done?

The individuals and teams that will contribute to intervention design should now explore the system, i.e. the individuals, items and processes working towards a common healthcare goal [11]. This will help focus the response and determine how best to use simulation expertise. QI utilises a number of different approaches and techniques that can facilitate understanding the system and inform the design of change, for instance process mapping. Simulation may be a powerful tool during process mapping [12], as it allows us to immerse ourselves in the process, allowing the integration of learning and change without disrupting clinical practice.

Once we have a better understanding of the system, the Model for Improvement guides us to identify what needs to be done by proposing 3 questions: What are we trying to accomplish? How will we know that a change is an improvement? What changes can we make that will result in an improvement? [13].

Consideration should be given to whether simulation is the appropriate tool to utilise following a key event or if a different approach might be more effective.

### How will simulation contribute?

Simulation can take many forms depending on location, modality and fidelity [14]. Before developing a simulation-based response, we should consider collaboratively how to best utilise simulation expertise. Simulation may assist with one or more of the following: testing the system, providing education and training (by itself or in the context of blended learning), or contributing to event debriefing, i.e. facilitation of shared reflective practice and system-focussed collaborative learning from the original key event.

- System testing simulation [15] allows us to explore existing or new processes and pathways, identifying latent threats before they lead to harm or evaluating ease of use of checklists and preparing for the use of new equipment or facilities. It may be carried out “in situ” (at the point of care) or in simulation facilities, depending on the circumstances.
- Simulation-based education and training may bridge gaps in knowledge, skills and attitudes and focus on technical abilities, application of drills, non-technical skills and/or team performance [16, 17].
- Debriefing is an inherent element of simulation-based education and training [18]. It consists of facilitated discussions guiding participants to reflect on simulated experiences. Debriefing conversations following real events in healthcare are referred to as clinical debriefing [19, 20]. This is a guided meeting during which teams discuss, interpret and learn from recent events.

Any intervention for change should be evaluated with due consideration to outcome, process and balancing measures [6].

### How should we share the learning?

Whilst following the above steps, learning can be derived from each stage of the framework as well as its entirety, i.e. from the key event, stakeholder analysis, greater understanding of the system through the use of improvement techniques, changes carried out and their evaluation.

This learning must be fed back to the teams and stakeholders involved as well as disseminated to the wider departments and organisation where appropriate, including patient safety teams. This might translate into organisational change, as lessons learned in the planning and delivery of the simulation intervention are adapted to different environments. Such interventions carry a risk of exacerbating the second victim phenomenon. It is therefore paramount to ensure the psychological safety of all individuals and teams involved during the trigger event [21].

## Discussion

It is rare that expertise in simulation and QI are available simultaneously. However, organisations are encouraged to integrate simulation-based methodologies into their safety management and quality improvement infrastructure, as well as identify opportunities for collaboration between safety management groups, quality improvement teams and education leads [22]. This framework supports all these teams in identifying events that can lead to the development of learning opportunities through simulation-based practice.

When simulation is used with a focus on improving healthcare processes and outcomes, it can be referred to as translational simulation [23]. Simulation for improvement can be classified as either proactive or reactive: the former includes its use for induction and training of new staff, prior to the introduction of new processes or innovations, for system redesign, or as part of routine system testing. Reactive simulation interventions comprise those following challenging situations with positive outcomes, identification of system weaknesses, near misses or critical events. Although the framework to guide simulation interventions following key events in healthcare focuses on the design of reactive simulation interventions, the principles described can also be applied to proactive simulation.

Whilst we have developed this guidance aiming to provide a practical process, we acknowledge that it might not be universally applicable due to local infrastructure or other relevant characteristics. Any comments on its usefulness, as well as any feedback on challenges to implementation or suggestions for its improvement, are welcome via the corresponding author.

## Conclusion

This guidance introduces applicable quality improvement principles, considers how simulation-based methodologies could be most beneficial in each context and presents a new way to combine QI and simulation approaches synergistically. Furthermore, it aligns with contemporary socio-technical models in healthcare, which emphasise the role of clinical teams in designing adaptation and change for improvement [10]. The psychology of change framework also highlights the importance of activating clinicians’ agency as a key element to advancing and sustaining collaborative improvement [5]. We believe that this guidance is a novel approach which will be of benefit to health and care teams preparing for, or responding to, adverse events, as well as promoting the concept of learning from everyday work.

Access to the full framework may be obtained via HEIW, ASPiH and SESAM websites. It can be downloaded from https://heiw.nhs.wales/files/improving-quality-through-simulation-framework/.

## Data Availability

Data sharing not applicable to this article as no datasets were generated or analysed during the current study.

## Abbreviations

ASPiH: Association for Simulation Practice in Healthcare
HEIW: Health Education and Improvement Wales
NHS: National Health Service, UK
PDSA: Plan-Do-Study-Act
QI: Quality improvement
SESAM: Society for Simulation in Europe
SEIPS: Systems Engineering Initiative for Patient Safety
